# Characterization of the putative polysaccharide synthase CpsA and its effects on the virulence of the human pathogen *Aspergillus fumigatus*

**DOI:** 10.1371/journal.pone.0216092

**Published:** 2019-04-26

**Authors:** Binita Nepal, Ryan Myers, Jessica M. Lohmar, Olivier Puel, Brett Thompson, Matthew Van Cura, Ana M. Calvo

**Affiliations:** 1 Department of Biological Sciences, Northern Illinois University, Dekalb, Illinois, United States of America; 2 Toxalim (Research Centre in Food Toxicology), Université de Toulouse, INRA, ENVT, INP-Purpan, UPS, Toulouse, France; Woosuk University, REPUBLIC OF KOREA

## Abstract

The fungus *Aspergillus fumigatus* is a ubiquitous opportunistic human pathogen capable of causing a life-threatening disease called invasive aspergillosis, or IA, with an associated 40–90% mortality rate in immunocompromised patients. Of the approximately 250 species known in the genus *Aspergillus*, *A*. *fumigatus* is responsible for up to 90% of IA infections. This study focuses on examining the role of the putative polysaccharide synthase *cpsA* gene in *A*. *fumigatus* virulence. Additionally, we evaluated its role in cellular processes that influence invasion and colonization of host tissue. Importantly, our results support that *cpsA* is indispensable for virulence in *A*. *fumigatus* infection of non-neutropenic hosts. Our study revealed that *cpsA* affects growth and sporulation in this fungus. Absence of *cpsA* resulted in a drastic reduction in conidiation, and forced overexpression of *cpsA* produced partially fluffy colonies with low sporulation levels, suggesting that wild-type *cpsA* expression levels are required for proper conidiation in this fungus. This study also showed that *cpsA* is necessary for normal cell wall integrity and composition. Furthermore, both deletion and overexpression of *cpsA* resulted in a reduction in the ability of *A*. *fumigatus* to adhere to surfaces, and caused increased sensitivity to oxidative stress. Interestingly, metabolomics analysis indicated that *cpsA* affects *A*. *fumigatus* secondary metabolism. Forced overexpression of *cpsA* resulted in a statistically significant difference in the production of fumigaclavine A, fumigaclavine B, fumigaclavine C, verruculogen TR-2, and tryprostatin A.

## Introduction

Invasive Aspergillosis (IA) is a life-threatening mycosis in immunocompromised individuals. The fungus *A*. *fumigatus* is the most common cause of IA [1], resulting in high mortality rates between 40% and 90%. The deadliness of *A*. *fumigatus* is in large part due to the small size of its conidia (2 to 3 μm in diameter). These spores can easily reach the lung alveoli, establishing an infection that can become angioinvasive, colonizing other organs in the human body, including the liver, kidneys, and brain. In the case of immunocompetent individuals, conidia are efficiently eliminated through normal mucociliary clearance. In addition, epithelial cells or alveolar macrophages are able to eliminate conidia and initiate a proinflammatory response that recruits neutrophils (PMN type). These neutrophils destroy hyphae from germinated conidia that evade macrophages. The highest risk of developing IA is primarily a consequence of a dysfunction of these defenses. In the immunocompromised, such as patients with hematological malignancies, transplants, prolonged steroid treatments and cancer and HIV, the systemic nature of the infection can be fatal [2,3,4]. The risk of IA increases due to neutropenia (depletion of neutrophils) and corticosteroid-induced immunosupression [5,6,7,8,9].

Among the factors that contribute to virulence are *A*. *fumigatus* cell wall integrity, the ability of the organism to adhere to surfaces and to rapidly adapt to environmental stresses encountered *in vivo*. Cell wall composition and integrity are important for survival of *A*. *fumigatus* in the host, as the cell wall protects the cell components from host defense systems [10]. In addition, it is known that the ability of an organism to adhere to surfaces is a precursor for biofilm formation [11]. Biofilms provide an important layer of protection from the host immune system [12]. Furthermore, when a microorganism encounters a variety of stressors, such as oxidative or osmotic stress, it must be able to adapt quickly in order to survive inside of the host environment [13]. Production of secondary metabolites could also influence *A*. *fumigatus* virulence [14]. This opportunistic pathogen is capable of producing up to 226 different secondary metabolites, some of these compounds can act as immunosuppressants, affecting pathogenetic processes [15].

Current IA treatment options are limited, usually consisting of a regimen of antifungal drugs including azoles, which inhibit the synthesis of ergosterol, and polyene drugs, which bind to ergosterol disrupting fungal cell membrane [16]. However, emerging isolates of *A*. *fumigatus* resistant to the current drug treatments have been identified [17]. Due to the devastating effects of IA on human and animal health, together with the emergence of drug-resistance, it is paramount to seek new strategies against these fungal infections, such as searching for novel genetic fungal elements that regulate virulence and/or factors that influence host colonization. These factors could serve as a possible genetic targets for the generation of novel antifungal drugs in the treatment of IA. One of these candidates, *cpsA*, encodes a putative polysaccharide synthase in *A*. *fumigatus*. A *cpsA* homolog was previously found in a mutagenesis screening in the model fungus *Aspergillus nidulans* [18]. In this model, *cpsA* is necessary for normal asexual and sexual development as well as for production of sterigmatocystin toxin and penicillin. *A*. *fumigatus* CpsA presents 73.2% sequence similarity and 66.2% identity with its putative homolog in *A*. *nidulans* [19]. Here we show that *A*. *fumigatus cpsA* influences multiple cellular processes in this opportunistic human pathogen. Specifically, our study indicated that *cpsA* is required for normal pathogenesis in a non-neutropenic murine model, in addition to being necessary for normal morphological development of *A*. *fumigatus*. *cpsA* was also found to be indispensable for normal cell wall composition and integrity, as well as for adherence to surfaces and resistance to oxidative stress. Furthermore, metabolomics analysis revealed that secondary metabolism is also *cpsA*-dependent.

## Materials and methods

### Strains and culture conditions

The *A*. *fumigatus* strains used in this study are listed in Table 1. Strains were grown on glucose minimal medium (GMM) [20] with the necessary supplements at 37°C. In the case of solid medium, 10 g/L agar was added. Fungal stocks were maintained in 30% glycerol at -80°C.

**Table 1:** Fungal strains used in this study.

Strain name	Pertinent genotype	Source
CEA10	Wild type (*veA+*)	Gift from Robert Cramer
CEA17	*pyrG1* (*veA*+)	Gift from Robert Cramer
TMNV1.1	*pyrG1*, Δ*cpsA*::*pyrG*^*A*.*para*^	This study
TBN1	Δ*cpsA*::*pyrG*^*A*.*para +*^, *cpsA*::*ptrA+*	This study
TBN3.1	*gpdA*::*cpsA*::*trpC*::*pyrG*, *pyrG1*, *veA+*	This study

### Construction of the *cpsA* deletion strain

In order to characterize the function of *cpsA* in *A*. *fumigatus*, a deletion strain was generated as follows. First, 1.1 kb 5’ UTR and 1.5 kb 3’ UTR fragments were PCR amplified from the *cpsA* locus using primers cps5F and cps5R, and cps3F and cps3R, respectively (all primers used in this study are listed in Table 2). The selectable marker, *pyrG*, was PCR amplified with cps-pyrGF and cps-pyrGR primers from *A*. *parasiticus* genomic DNA. Fusion PCR [21] was carried out using P7-F(cps5nest) and P8-R(cps3nest) primers resulting in a 5025 bp deletion cassette. The fusion cassette was then transformed into the CEA17 strain. Genomic DNA from the transformants was analyzed by Southern blot. The selected deletion mutant was designated as TMNV1.1.

**Table 2:** Primers used in this study.

Name	Sequence (5’→ 3’)
cps5F cps5R	CCCGTGCTGACAGCTGACAGTAG GATTAACGACCGGCCAACGAAGTT T
cps3F cps3R	TTGGGGTTCTGTTCTTCCCATGCTC GGGCAGTGATAGGATGGGCAAC
cps-pyrGF	GATTAACGACCGGCCAACGAAGTTTACCGGTCGCCTCAAACAATGCTCT
cps-pyrGR	GAGCATGGGAAGAACAGAACCCCAAGTCTGAGAGGAGGCACTGATGCG
P7-F(cps5nest)	GTCCCTAAGCTCGCTTCTCCTAGC
P8-R(cps3nest)	GGAGAGAACTGAAATGGCGGCTC
1925F	TAAATTGCGGCCGCTTCTGTGAGATGGCATGAAAAGTTCGA
1926R	AAAAAAGCGGCCGCACCAATAGTTGTTAAGACCCCTCCAC
1918F	AAAAAAGGCGCGCCATGGCTTTCCCATTCATGCGAG
1986R	AAAAAAGGCGCGCCATGGCTTTCCCATTCATGCGAG
592-gpdaF	AAGTACTTTGCTACATCCATACTCC
1927R	TTATTGGCGCGCCTCATTGAGTTATGAAGTTGGGGTAGTACG
849 18S-qRtPCR-F	TAGTCGGGGGCGTCAGTATTCAGC
850 18S-qRtPCR-R	GTAAGGTGCCGAGCGGGTCATCAT
1919 cpsA-qRT-F	GGTGAGGTTGCTTCTCCAGTTGC
1920 cpsA-qRT-R	GATGGTATCACACGGCTGGGAGA
1158 brlA-qRT-F	TGCACCAATATCCGCCAATGC
1159 brlA-qRt-R	CGTGTAGGAAGGAGGAGGGGTTACC
2105 qPCR abaA F 2106 qPCR abaAR	GTCAGCAAAGCCGAAGATGGACTACC GTTGTCGTGGCTCAAGGCGTAC

### Generation of the *cpsA* complementation strain

The complementation vector was generated as follows: A DNA fragment of 4.9 kb containing the *cpsA* coding region and 1.4 kb 5’ UTR and 3’ UTR regions was PCR amplified with primers 1925F and 1926R with engineered *Not*I sites. The PCR product was then digested with *Not*I and ligated to the vector pTD3, previously digested with the same enzyme. pTDS3 harbors the *Aspergillus oryzae* pyrithiamine resistance gene (*ptrA*) used as selectable marker in fungal transformation. The ligation resulted in the complementation vector pBN1. This plasmid was then transformed into the Δ*cpsA* strain, TMNV1.1. The transformants were confirmed by diagnostic PCR using primers 1918F and 1986R. The selected *cpsA* complementation strain (Com) used in this study was designated as TBN1.

### Construction of the overexpression *cpsA* strain

To generate an overexpression strain (OE), the *cpsA* coding region was first PCR amplified with primers 1918F and 1986R, which contain engineered *Asc*I and *Not*I restriction sites. The DNA fragment was then digested with *Asc*I and *Not*I and ligated to the pTDS1 vector previously digested with the same enzymes. pTDS1 contains the constitutive *gpdA*(*p*) promoter and *trpC*(*t*) terminator sequences in addition to the *A*. *fumigatus pyrG* marker. The resulting vector, pBN2, was then transformed into CEA17. Transformants were confirmed using diagnostic PCR with primers 592-gpdaF and 1927R. The selected overexpression strain used in this study was designated as TBN3.1.

### Mouse model pathogenicity analysis

Pathogenicity studies were carried out as previously described by Myers et al. [22] with minor modifications. Briefly, six-week old female, outbred ICR Swiss mice, weighing approximately 25 g were used for this experiment. Fifty mice divided into 5 separate groups were used, each group contained 10 mice. Animals were rendered neutropenic by intraperitoneal injection of cyclophosphamide (150 mg/kg of body weight) on days -4, -1 and 3 days post infection and Kenalog (40 mg/kg) on the day of infection. The immunosuppressed mice were infected with fungal spores of *A*. *fumigatus* CEA10 wild type, Δ*cpsA*, and Com strains. Sedated mice (10 mice per strain) were infected by nasal instillation of 10^6^ spores/40 μl of PBS. Post infection mice were observed three times daily for first five days and 5 times on day six and seven. All remaining mice were euthanized on day eight.

A separate experiment was carried out using a non-neutropenic model. Mice were treated with Kenalog (40 mg/kg) subcutaneously one day prior the infection. The animals were then infected intranasally with 10^6^ spores/ 40 μl PBS. The mice were monitored 3 times daily for 14 days. All remaining mice were euthanized on day 15. Statistical analysis for this and the previous experiments was done using IBM SPSS software. P-values lower than 0.05 was determined to be statistically significant.

This study was carried out in strict accordance with the Guide for the Care and Use of Laboratory Animals of the National Research Council. The protocol was approved by the Institutional Animal Care and Use Committee of Northern Illinois University (Permit #12–0006). All efforts were made to minimize suffering. Humane euthanasia by CO_2_ inhalation was performed when mice met criteria indicating a moribund state; these endpoints include behaviors of unresponsiveness to tactile stimuli, inactivity, lethargy, staggering, anorexia and/or clinical signs of bleeding from the nose or mouth, labored breathing, agonal respirations, purulent exudate from eyes or nose, abnormally ruffled fur, or greater than 20% weight loss. The method of euthanasia by CO_2_ inhalation is consistent with recommendations of the Panel on Euthanasia of the American Veterinary Medical Association.

### Morphological analysis

The *A*. *fumigatus* CEA10 wild type, Δ*cpsA*, complementation (Com) and *cpsA* overexpression (OE) strains were point-inoculated on GMM and incubated in the dark at 37°C for 64 h. Effects on vegetative growth were assessed as colony diameter in mm. The experiment was performed with three replicates.

To determine whether *cpsA* is involved in the regulation of conidiation in *A*. *fumigatus*, the strains were top-agar inoculated on GMM, and incubated in the dark at 37°C for 72 h. Then, 7 mm-diameter cores were harvested and homogenized in water. Conidia were quantified using a hemocytometer (Hausser Scientific, Horsham, PA) and a Nikon Eclipse E-400 bright-field microscope (Nikon Inc., Melville, NY). The experiment was carried out in triplicate.

### Determination of cell wall defects

To assess whether *cpsA* affects the *A*. *fumigatus* cell wall, the wild type, Δ*cpsA*, Com, and OE strains were exposed to different concentrations of sodium dodecyl sulphate (SDS) as previously described [22] with minor modification. Briefly, the wild type, Δ*cpsA*, Com, and OE strains were point- inoculated on GMM supplemented with 0%, 0.005%, 0.01%, 0.015% and 0.02% SDS. The cultures were incubated at 37°C for 72 h under dark condition, when they were photographed.

An additional experiment was carried out in which the *A*. *fumigatus* strains were point-inoculated on GMM supplemented with 32 μg/mL and 64 μg/mL of Nikkomycin Z in a 24-well plate. The cultures were incubated at 37°C for 48 h and photographed.

### Cell wall chemical analysis

Previously, the *A*. *nidulans cpsA* was found to regulate cell wall composition by affecting the levels of central cell wall components such as mannoprotein, glucan and chitin [19]. Due to these results it is possible that *cpsA* may also regulate cell wall composition in *A*. *fumigatus*. To determine the levels of mannoprotein, glucan and chitin in the wild type, Δ*cpsA*, Com and OE strains, a protocol previously described [19] was utilized with minor modifications. Briefly, the *A*. *fumigatus* strains were inoculated into 50 mL of liquid GMM medium (10^6^ spores/ mL) and incubated at 37 °C for 48 h at 250 rpm in a rotary shaker. Mycelium was harvested using miracloth (Calbiochem, San Diego, CA), washed three times with sterile ddH_2_O and stored at -20 °C. Prior to analysis, mycelia were treated with 1 ml of cell wall buffer (2% SDS in 50 mM Tris-HCl buffer supplemented with 100 mM Na-EDTA, 40 mM β-Mercaptoethanol and 1 mM PMSF) and boiled for 15 minutes to remove any unbound cell wall proteins and water soluble sugars. Then, the buffer was removed and mycelia were washed 3 times with sterile ddH_2_O. The samples were then lypholized overnight. Approximately, 40 mg of lyophilized mycelia per strain was used for the analysis, including 3 replicates per strain. The samples were treated with 3% NaOH at 75 °C for one hour and then centrifuged at 15,000 g for 15 minutes to separate the soluble and insoluble fractions. The supernatant was collected and used for the analysis of mannoprotein and any soluble glucans that may be present. The pellet was further digested with 96% formic acid for 4 h at 100 °C. After digestion, the formic acid was evaporated and the residue re-suspended in 1 mL of sterile ddH2O which was further used to analyze the amount of chitin and insoluble glucan present. Mannoprotein, glucan, and chitin levels were determined as previously described [23, 24, 25]. Mannoprotein, glucan, and chitin levels were determined by absorbance at 560 nm, 490 nm, and 520 nm, respectively, using an Epoch spectrophotometer (Biotek, Winooski, VT).

### Adhesion assay

To evaluate the possible role of *cpsA* in adhesion capacity, each strain was inoculated in 50 ml GMM at a concentration of 10^6^ spores/ml. Aliquots of 130 μl from these suspensions were added to each well of a 96-well plate and incubated at 37°C for 24 h. After incubation, the mycelial mat and medium was removed. The remaining biomass adhered to the walls of the plastic wells was washed three times with water before staining with 130 μl 0.01% Crystal Violet (CV) in water for 20 min at room temperature. The stained wells were washed three times with water, allowed to dry, and then destained with 130 μl of 30% acetic acid. The absorbance was read at 560 nm on an Epoch spectrophotometer (Biotek, Winooski, VT).

### Environmental stress tests

To assess the role of *cpsA* in the resistance to oxidative stress, the wild type, Δ*cpsA*, Com and OE strains were point-inoculated on solid GMM supplemented with 0, 10, 20, 30, 40 and 50 μM menadione in a 24-well plate and incubated at 37 °C for 72 h. The experiment included two replicates.

To evaluate the resistance of the *A*. *fumigatus* strains to osmotic stress, the strains were point- inoculated on GMM supplemented with 0.8 M Sucrose, 1.2 M Sorbitol, 0.6 M KCl, or 0.7 M NaCl. The cultures were incubated at 37°C, for 72 h in the dark.

### Metabolomics analysis

#### Production, collection, and extraction of secondary metabolite compounds

The *A*. *fumigatus cpsA* strains were inoculated at a concentration of 10^6^ spores/ml into 250 ml flasks containing 50 ml of liquid YES medium (yeast extract 20 g/L and sucrose 40 g/L, pH 5.8 ± 0.2) and incubated at 37°C at a speed of 250 rpm for 5 days. The experiment was carried out using 4 replicates for each fungal strain. After 5 days, 40 ml of supernatant was collected by filtering through miracloth (Calbiochem, San Diego, CA). Secondary metabolites were extracted using a 1:1 chloroform to supernatant ratio. The mix was vortexed vigorously and left overnight for separation. The chloroform layer was collected and transferred to a 50 ml beaker where it was allowed to evaporate. The extracts were then resuspended in 500 μl of methanol and filtered through a 0.2 μm filter before being allowed to evaporate again prior to resuspension before the LC-MC analysis.

#### Liquid chromatography coupled with mass spectrometry (LC-MS) analysis

Sample analysis was performed using high performance liquid chromatography (HPLC) coupled to an LTQ Orbitrap XL high resolution mass spectrometer (HRMS) (Thermo Fisher Scientific, Les Ulis, France). Briefly, extracts were dissolved in 500 μl of water-acetonitrile and 10 μL of this suspension were injected into a reversed phase (150*2.0 mm) 5 μm Luna C18 (2) column (Phenomenex, Torrance, CA, USA) operated at a flow rate of 0.2 mL/min. A gradient program was performed with 0.05% formic acid (phase A) and 100% acetonitrile (phase B) with the following elution gradient: the elution started with a linear gradient ranging from 20% to 50% in 30 min, then phase B was increased to 90% within 5 min. After a 10 min isocratic elution, the gradient was decreased to initial value within 5 min and remained at this value for the last 10 min. HRMS acquisitions were achieved with electrospray ionization (ESI) in the positive and negative modes as follows: spray voltage +5.5 kV, capillary temperature 350°C, sheath gas (N_2_) flow rate 30 au (arbitrary units), auxiliary gas (N_2_) flow rate 10 au in the positive mode, and spray voltage -3.7 kV, capillary temperature 350°C, sheath gas (N_2_) flow rate 30 au, auxiliary gas (N_2_) flow rate 10 au in the negative mode. Full MS spectra were acquired at a resolution of 7500 with a range of mass-to-charge ratio (*m/z)* set to 50–850 while the MS/MS spectra were generated at low resolution by collision-induced dissociation (CID) according to the following parameters: collision energy = 35 eV, isolation width = 1.5 Da, activation Q = 0.250 and activation time = 30 min. The identity of compounds was confirmed by comparison either with HPLC-MS^2^ analysis of a standard compound or on a basis of results obtained in [26, 27]. The chaetominine standard was purchased from Bioaustralis (Smithfield, Australia).

### Gene expression analysis

Plates containing 30 mL of liquid GMM were inoculated with 10^6^ spores/mL of *A*. *fumigatus* wild type, Δ*cpsA*, Com and OE strains, and incubated in the dark at 37 °C, for 48 h and 72 h. Total RNA was extracted using TriSure. Approximately 5μg of extracted RNA was then treated with DNase and 1 μg of treated RNA was then reverse transcribed using Moloney murine leukemia virus (MMLV) reverse transcriptase (Promega). Synthesized cDNA was used to evaluate the expression of *cpsA*, *brlA* and *abaA* by qRT- PCR with the Applied Biosystems 7000 Real -Time PCR system. SYBR green jumpstart TAq Ready Mix (Sigma) was used for fluorescence detection. The primer pairs used for qRT-PCR are also listed in Table 2. The relative expression levels were calculated using the 2^-ΔΔCT^ method [28].

### Statistical analysis

Statistical Analysis was carried out for all quantitative data in this study unless specified differently. ANOVA (Analysis of Variance) in conjunction with Tukey’s post hoc test was performed using the statistical software program R version x64 3.3.0.

## Results

### Identification of *A*. *fumigatus cpsA*

Previously, a *cpsA* homolog was found through a random mutagenesis study in *A*. *nidulans* (Ramamoorthy et al., 2012). Putative *cpsA* homologs are present in numerous fungal species [19, 29, 30]. A BLASTp search was performed with the *A*. *nidulans* CpsA amino acid sequence to look for homologs in *A*. *fumigatus*. From this search, the best hit was XP_746682, annotated as a putative polysaccharide synthase. This protein has an identity score of 83% with respect to CpsA in *A*.*nidulans*. To confirm our results, we performed a reciprocal BLAST and found that XP_746682 best hit in *A*. *nidulans* was CpsA (AN9069).

### Absence of *cpsA* results in decreased virulence in a non-neutropenic murine model

*Aspergillus fumigatus* is the most common cause of IA [2, 31]. The increasing rate of organ transplant and stem cell transplant, preexisting lung infections such as cystic fibrosis, sarcoidosis, steroid therapy or any condition that weakens the immune status are major predisposing factors for IA [32]. Since *A*. *nidulans cpsA* influences multiple cellular processes in *A*. *nidulans* [19], we tested whether *A*. *fumigatus cpsA* is involved in virulence in two different immunosuppressed environments, neutropenic and non-neutropenic mice. First, the strains for these experiments were generated as described in the Material and Methods section. The Δ*cpsA* strain was confirmed by Southern analysis (S1A Fig), obtaining the expected 2.1 kb band for Δ*cpsA* and 5.6 kb for the wild type sample, indicating successful replacement of the *cpsA* coding region by the *A*. *parasiticus pyrG* selection marker. In addition, the *cpsA* complementation was obtained and confirmed using diagnostic PCR (S1B Fig).

**Fig 1:**
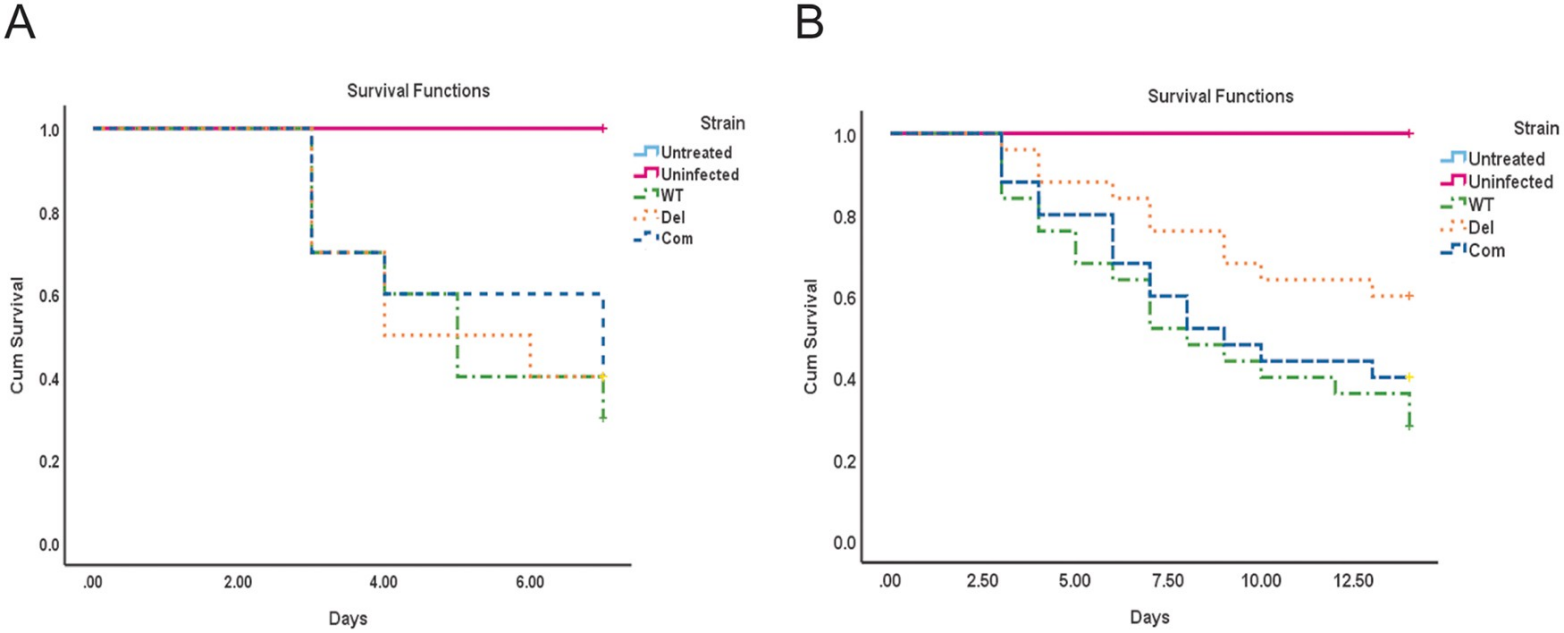
Effect of *cpsA* in virulence in a murine model. The *A*. *fumigatus* wild type (WT), Δ*cpsA* and Com strains were used to infect female ICR outbred mice. (A) For the neutropenic model, mice were treated with intraperitoneal injection of cyclophosphamide and Kenalog as described in Materials and Methods section. Untreated controls did not receive any treatment and uninfected controls received both cyclophosphamide and Kenalog treatment but not fungal spores. Post infection mice were observed three times daily for first five days and 5 times on day six and seven. Presented data is the result of single experiment (N = 10 total for each group). (B) For the non-neutropenic model, mice were treated only with Kenalog, one day prior to the infection. Untreated controls did not receive Kenalog treatment, and uninfected controls received the Kenalog treatment but not fungal spores. Mice were monitored for survival for 14 days. Statistical analysis was done using IBM SPSS statistics 25. Presented data are the combined results of two independent experiments (N = 25 total for each groups).

Our virulence experiment with neutropenic mice showed that *cpsA* is dispensable in this immune-depressed environment (Fig 1A), however when the mice were immune-depressed with corticosteroids only, a statistically significant difference in virulence was observed depending on the presence or absence of *cpsA* (Fig 1B). The groups infected with spores from the deletion *cpsA* mutant showed higher survival rate when compared to the groups infected with wild -type spores. The Com strain showed a similar virulence pattern to that of the wild-type strain, indicating rescue of the wild-type phenotype when the *cpsA* wild-type allele was reintroduced in the deletion mutant. No mortality was reported in the control groups.

### *cpsA* is required for normal growth and conidiation in *A*. *fumigatus*

**Fig 2:**
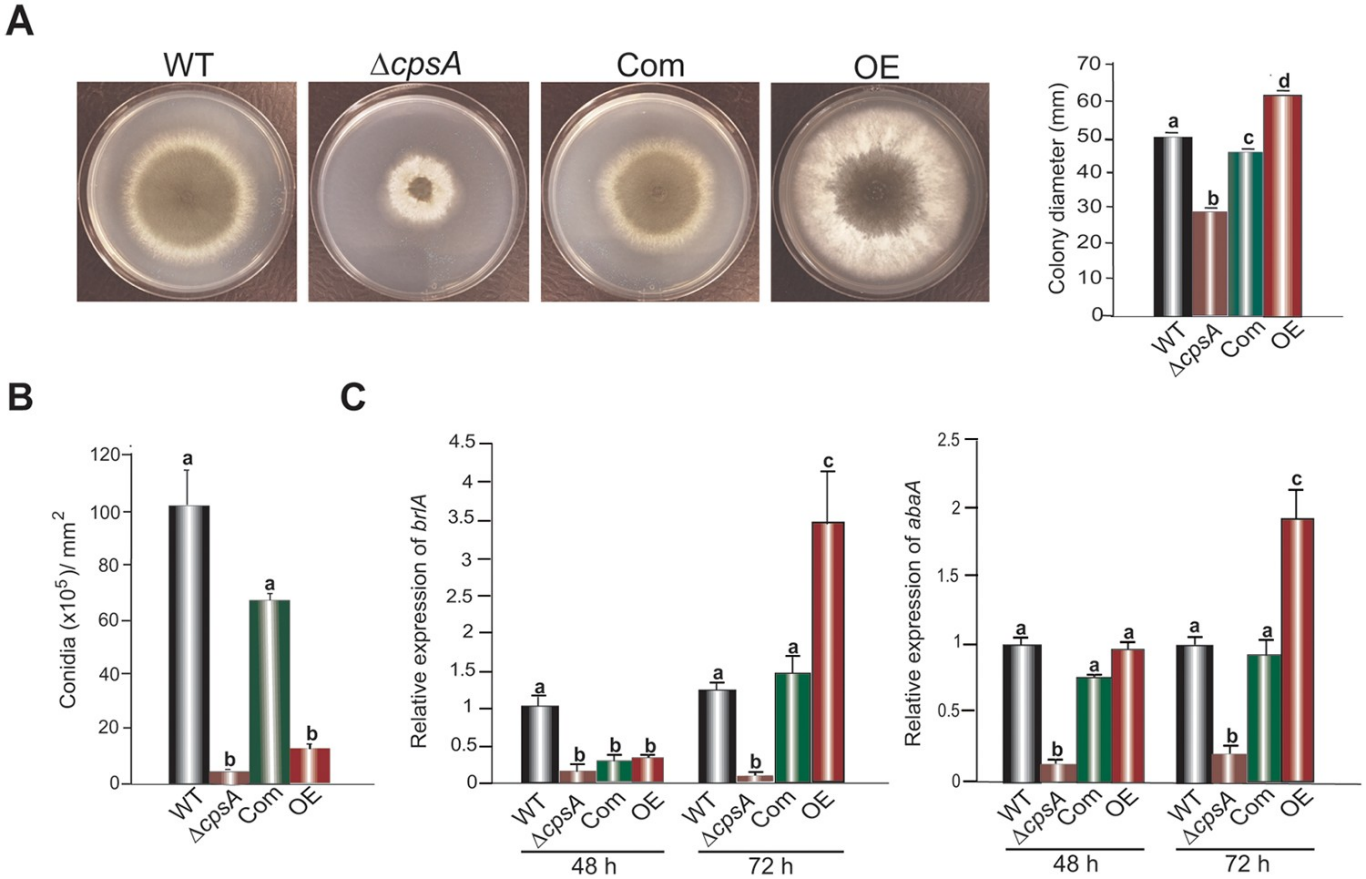
*cpsA* influences growth and conidiation in *A*. *fumigatus*. (A) Point-inoculated cultures of *A*. *fumigatus* wild type, Δ*cpsA*, Com and OE strains. Plates were incubated at 37°C for 48 h. On right, measurement of colony diameter. (B) Conidial quantification of *A*. *fumigatus* wild type, Δ*cpsA*, Com and OE top-agar inoculated cultures grown at 37°C for 72 h. (C) Gene expression analysis of *brlA* and *abaA* using qRT-PCR from mycelia collected from liquid stationary cultures incubated in the dark for 48 h and 72 h. The relative expression was calculated using 2^-ΔΔCT^ method as described by Livak and Schmittgen [28]. Values were normalized to the expression level of WT at 48 h. The error bar represents the normalized standard error.

To determine whether *cpsA* regulates morphological development in *A*. *fumigatus*, the wild type, Δ*cpsA*, Com and OE strains were point-inoculated on GMM and incubated for 64 h. The OE strains included in this experiment were confirmed by diagnostic PCR (Fig 1C). Absence of *cpsA* resulted in a significant decrease in vegetative colony growth compared to the controls, whereas forced over-expression of *cpsA* resulted in an increase in colony growth and aerial mycelium (Fig 2A). Complementation with the *cpsA* wild-type allele rescued wild-type phenotype.

Conidiation is an efficient dispersal mechanism in *A*. *fumigatus* [2]. Previously, *cpsA* was found to regulate asexual development in *A*. *nidulans* [19]. In order to investigate the role of *cpsA* in asexual development in *A*. *fumigatus*, spores from wild type, Δ*cpsA*, Com and OE strains were top-agar inoculated on GMM and incubated for 72 h. The Δ*cpsA* strain showed a statistically significant reduction in conidial production compared to the wild type. Interestingly, *cpsA* OE strain also showed a reduction in conidiation levels when compared to the isogenic control strains (Fig 2B). Gene expression analysis of *brlA* and *abaA*, both transcription factor genes of the central regulatory pathway controlling conidiophore formation in Aspergillus [33,34] revealed downregulation of both genes in the absence of *cpsA* in 48 h and 72 h old cultures (Fig 2C). Differently from Δ*cpsA*, the OE *cpsA* strain presented a significant increase in *brlA* and *abaA* after 72 h of incubation (Fig 2C).

### *cpsA* is necessary for normal cell wall integrity and composition

**Fig 3:**
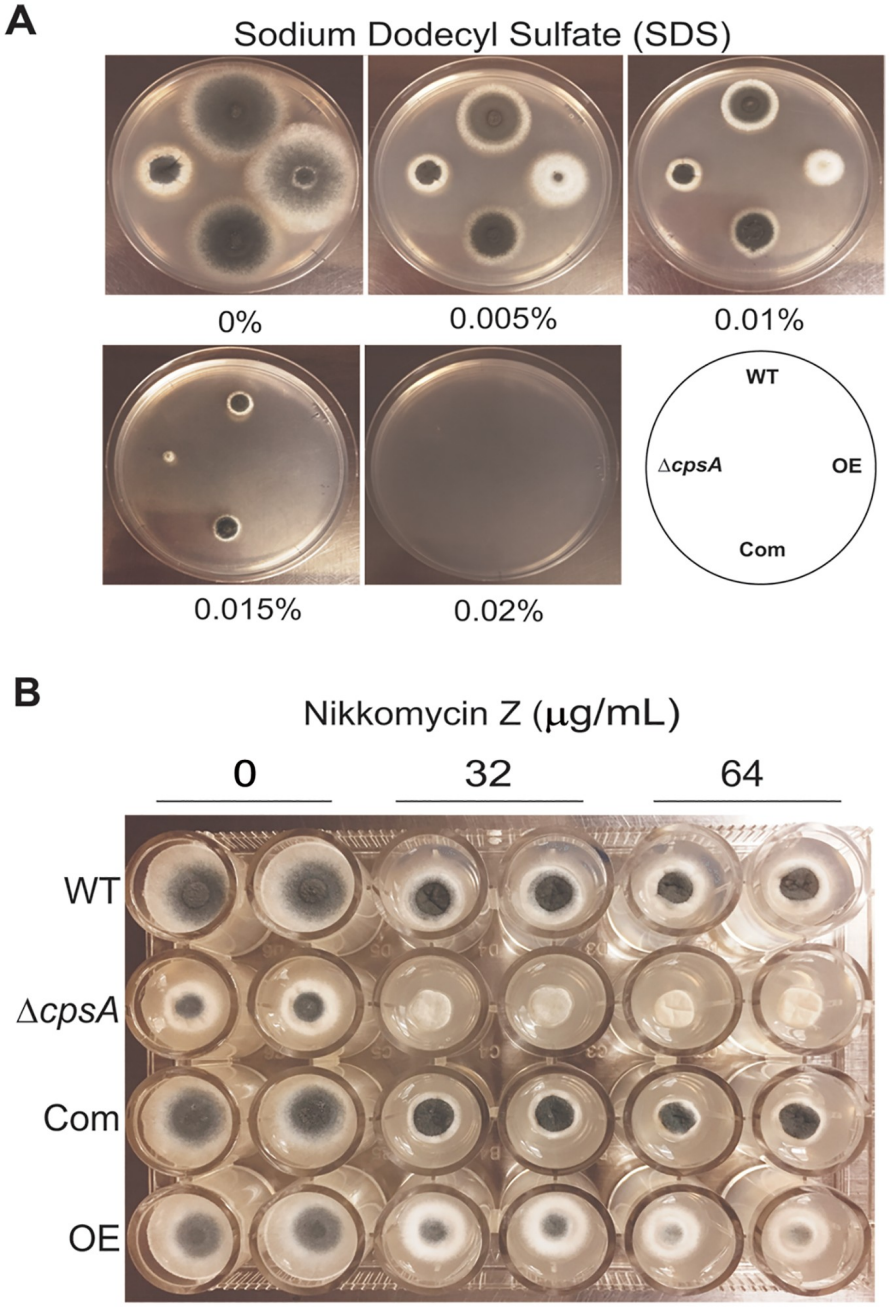
Cell wall stress assay. A) *A*. *fumigatus* wild type, Δ*cpsA*, Com and OE strains were point-inoculated on GMM supplemented with different concentration of SDS and incubated at 37°C for 72 h B) The above *A*. *fumigatus* strain set was point-inoculated on GMM supplemented with the indicated concentrations of Nikkomycin Z in a 24-well plate and incubated for 24 h at 37°C.

Fungal cell wall is a rigid structure that constitutes a protective barrier against environmental stresses. Its integrity is essential for fungal survival [10]. Our study revealed that *cpsA* also affects cell wall integrity in this opportunistic human pathogen. Both Δ*cpsA* and OE *cpsA* showed increased sensitivity when exposed to SDS in the culture medium. A concentration of 0.015% significantly reduced the growth of Δ*cpsA* in comparison to the isogenic control strains (Fig 3A). In addition, when grown in the presence Nikkomycin Z, a chitin synthase inhibitor, the deletion strain presented a completely aconidial phenotype. However, no noticeable difference was observed in colony radial growth (Fig 3B).

The results from the SDS and Nikkomycin Z analyses suggest that lack of *cpsA* could cause cell wall defects. To further evaluate this possibility the cell wall chemical composition was analyzed in the wild type, Δ*cpsA*, Com and OE strains. Specifically, we found that deletion of the *cpsA* gene in *A*. *fumigatus* caused a statistically significant increase in mannoprotein levels. Glucan levels present in the soluble alkali of Δ*cpsA* were similar to those in the wild type, however, a statistically significant increase in glucan levels in the insoluble alkali was observed. Chitin levels were also evaluated, and our results indicated that *cpsA* does not affect chitin biosynthesis in *A*. *fumigatus*. Forced over-expression of *cpsA* did not alter cell wall composition (Table 3).

**Table 3:** Cell wall composition (μg/mg ± standard error).

	Alkali-Soluble		Alkali-Insoluble	
	Mannoprotein	Glucan	Glucan	Chitin
WT	58.88 ± 4.12 A	183.98 ± 3.27 A	87.62 ± 9.75 A	4.89 ± 0.52 A
Δ*cpsA*	83.04 ± 5.98 B	229.49 ± 10.23 A	239.07 ± 24.52 B	7.14 ± 1.35 A
Com	62.65 ± 2.57 A	268.71 ± 27.13 A	140.72 ± 7.28 A	6.94 ± 0.95 A
OE	53.51 ± 4.10 A	243.50 ± 28.51 A	89.07 ± 16.00 A	5.78 ± 0.67 A

### *cpsA* affects adhesion to surfaces

**Fig 4:**
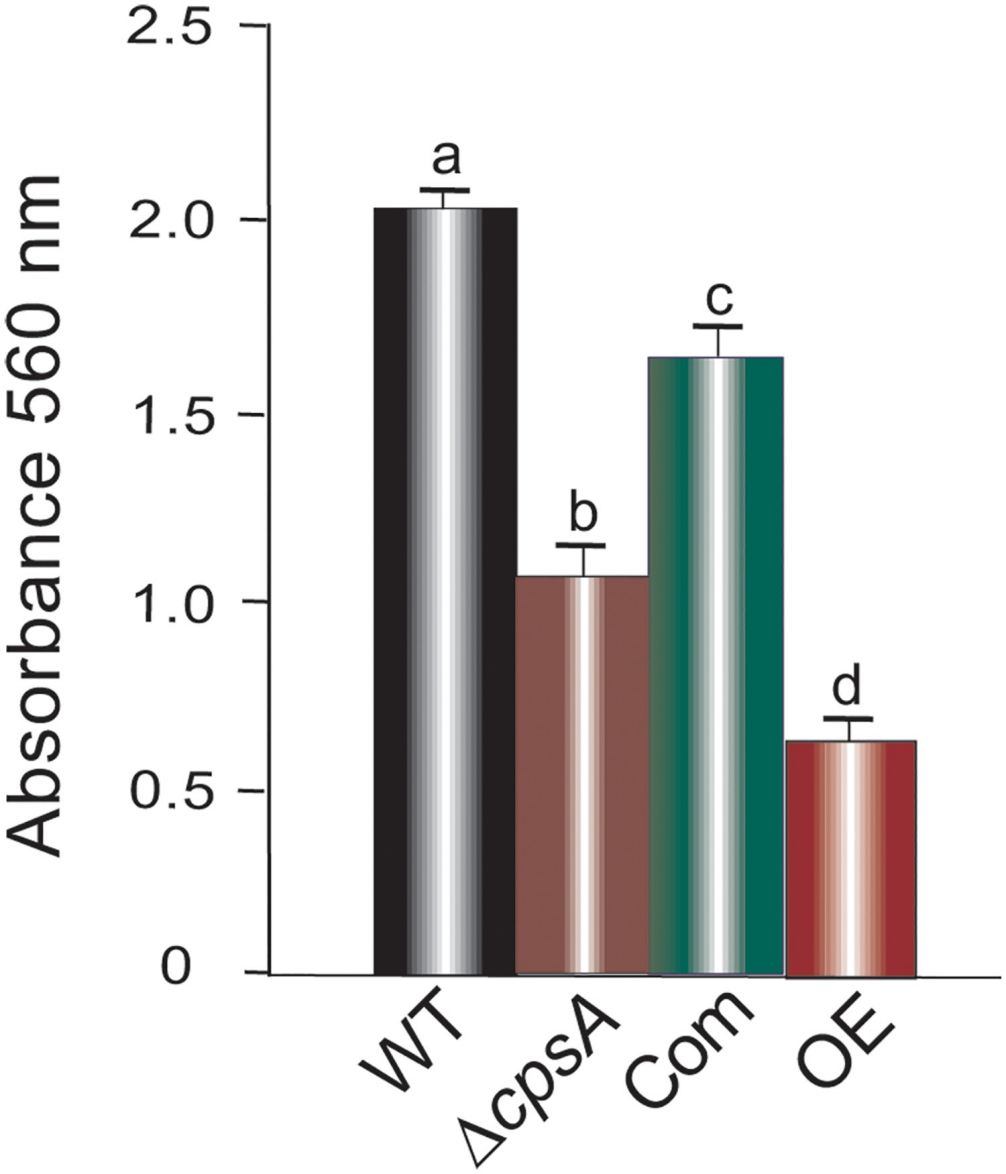
*cpsA* affects adhesion capacity. Liquid GMM (130μl) where inoculated with 10^6^spores/mL of wild type, Δ*cpsA*, Com and OE strains in individual wells of a Polystyrene 96-well culture plate. Twenty-four wells were used per strain. Mycelium was stained with Crystal violet as described in Materials and Methods section. The plate was incubated at 37°C for 24 h. The bar graph represents the average OD for each strain measured at 560 nm. The error bar represents the standard error.

Fungal biofilm is crucial for fungal pathogenicity. *A*. *fumigatus* produces biofilm on both biotic and abiotic surfaces [35]. One requirement for biofilm formation is the ability of an organism to adhere to a surface [11]. To examine the effect of *cpsA* on adherence we inoculated liquid GMM with fungal spores from the wild type, Δ*cpsA*, Com, and OE strains in a 96 well plate. Our analysis revealed that either absence or forced overexpression of *cpsA* resulted in a slight but significant decrease in adhesion capacity in 24 h old cultures (Fig 4).

### *cpsA* influences resistance to oxidative stress and is dispensable for resistance to osmotic stress

**Fig 5:**
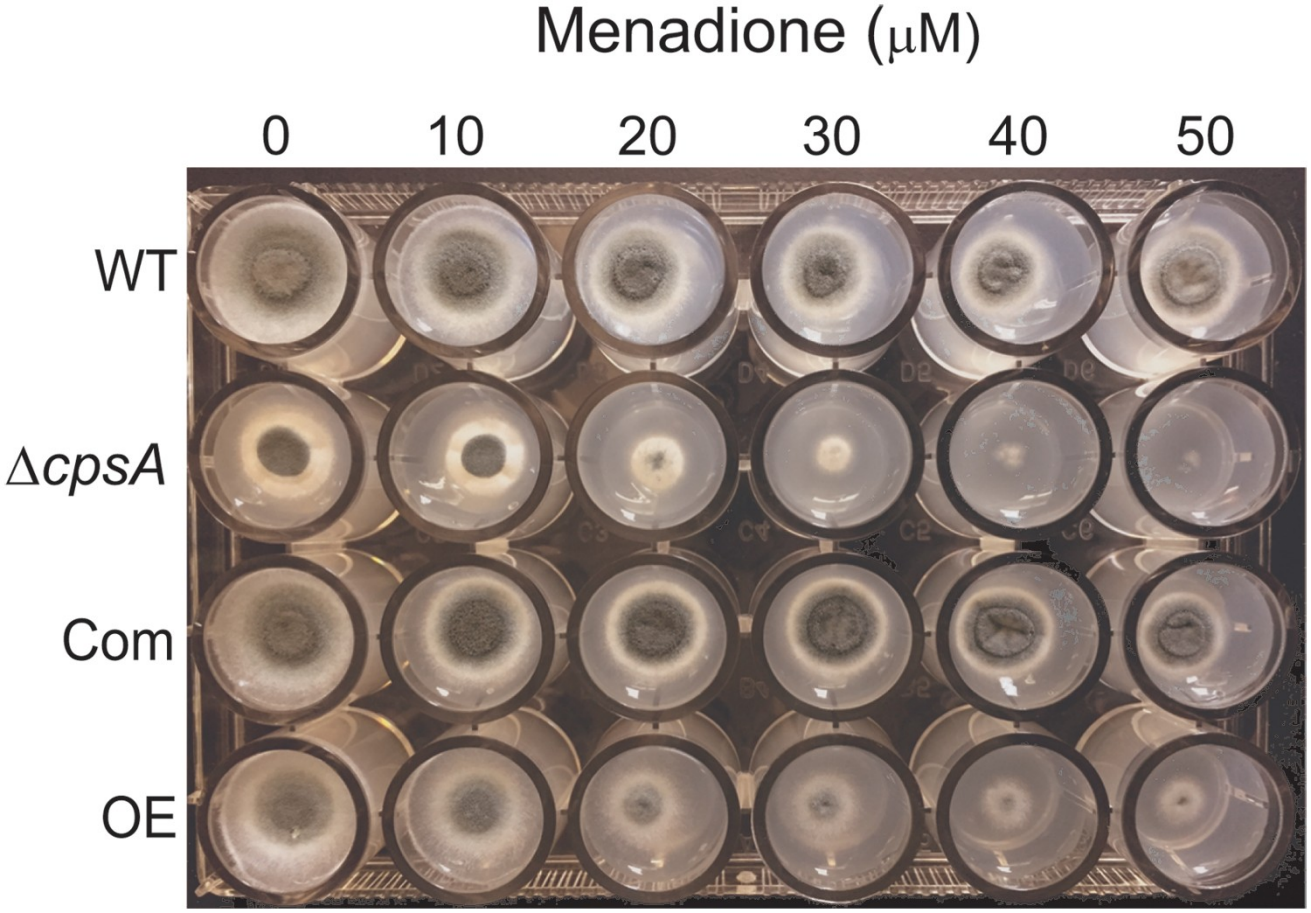
*cpsA* is required for normal resistance to oxidative stress. A 24-well plate containing GMM supplemented with different concentrations of menadione, was point-inoculated with the *A*. *fumigatus* wild type, Δ*cpsA*, Com and OE strains and incubated at 37 °C for 72 h.

Airborne conidia are inhaled and deposited in bronchioles and alveolar spaces, where the organism may be exposed to reactive oxygen species (ROS). ROS cause damage to various cellular components such as lipids, proteins and DNA. Oxidative stress resistance plays a vital role in protecting cells from possible damage caused by ROS [36]. We examined the role of *cpsA* in resistance to oxidative stress exposing the wild type, Δ*cpsA*, Com and OE strains to different concentrations of Menadione. Growth of Δ*cpsA* was completely abolished at a concentration of 50 μM (Fig 5). In addition, overexpression *cpsA* showed a marked reduction in growth at this concentration although colonies still formed.

In this study we also examined the possible role of *cpsA* in osmotic stress resistance supplementing GMM with different osmotic stress reagents such as 0.6 M Potassium chloride (KCl), 0.7 M Sodium chloride (NaCl), 0.8 M Sucrose and 1.2 M Sorbitol. When grown in presence of KCl and NaCl no difference in growth between the deletion mutant and the wild type control was detected (S2 Fig). In addition, when grown on GMM the OE *cpsA* strain normally presents an increase in colony growth when compared to the wild type strain, however, grown on GMM supplemented with KCl or NaCl the colony diameter was similar to that of the wild type. We noted that when grown on medium supplemented with Sucrose and Sorbitol the Δ*cpsA* strain produced a dark green pigment visible on the back of the colony that was absent in the control strains.

### *cpsA*-dependent metabolomics analysis

Previously *cpsA* was found to be necessary for the production of several secondary metabolites in the model organism *A*. *nidulans* [19]. We hypothesize that *cpsA* would also regulate secondary metabolism in *A*. *fumigatus*. To test this hypothesis, the *A*. *fumigatus* wild type Δ*cpsA*, Com and OE strains were grown in liquid stationary YES medium for 5 days. Our LC-MS analysis revealed statistically significant decreases in fumigaclavine A, fumigaclavine B, fumigaclavine C along with statistically significant increases in verruculogen TR-2 and tryprostatin A in the OE *cpsA* strain compared to the wild type (Table 4). Interestingly, the production of fumigaclavine A and C were unaffected in the absence of the *cpsA* gene, whereas the production of fumigaclavine B was increased in the deletion *cpsA* strain. In addition, the production of verruculogen TR-2 and tryprostatin A were not altered in the absence of *cpsA* (Table 4).

**Table 4:** LC-MS analysis data (average of normalized values ± standard error).

Compound	WT	Δ*cpsA*	Com	OE *cpsA*
**Fumigaclavine A**	**2.40 x 10**^**7**^ **± 8.6 x 10**^**6**^	**5.4 x 10**^**7**^ **± 1.7 x 10**^**7**^	**5.1 x 10**^**7**^ **± 2.2 x 10**^**6**^	**1.44 x 10**^**6**^ **± 6.8 x 10**^**5**^
**Fumigaclavine B**	**6.0 x 10**^**7**^ **± 4.3 x 10**^**7**^	**3.3 x 10**^**8**^ **± 1.3 x 10**^**8**^	**5.8 x 10**^**7**^ **± 1.8 x 10**^**8**^	**Not detected**
**Fumigaclavine C**	**1.8 x 10**^**8**^ **± 1.5 x 10**^**7**^	**3.5 x 10**^**8**^ **± 5.7 x 10**^**8**^	**4.9 x 10**^**8**^ **± 2.3 x 10**^**8**^	**1.9 x 10**^**7**^ **± 5.7 x 10**^**6**^
**TR2**	**1.2 x 10**^**7**^ **± 1.8 x 10**^**6**^	**9.7 x 10**^**6**^ **± 2.1 x 10**^**6**^	**2.3 x 10**^**7**^ **± 5.3 x 10**^**6**^	**5.5 x 10**^**7**^ **± 1.1 x 10**^**7**^
**Tryprostatin A**	**5.1 x 10**^**7**^ **± 1.8 x 10**^**7**^	**7.0 x 10**^**7**^ **± 1.6 x 10**^**7**^	**5.5 x 10**^**7**^ **± 5.6 x 10**^**6**^	**2.8 x 10**^**8**^ **± 2.2 x 10**^**7**^

## Discussion

In fungi, polysaccharides play a crucial role in structural integrity, and comprise more than 90% of the cell wall [37]. Besides structural roles, fungal polysaccharides serve as an energy reserve in the form of intracellular inclusions [38]. Polysaccharides also play a role in pathogenesis by influencing the host-pathogen interaction [39]. *cpsA*, annotated as a putative polysaccharide synthase gene, was identified in a mutagenesis screening in the model fungus *A*. *nidulans* where it influenced several cellular processes including development and secondary metabolism [18,19]. Sequence analysis showed that *cpsA* homologs exist in other fungal species including the opportunistic human pathogen *A*. *fumigatus*.

Although *A*. *fumigatus* is a saprophytic fungus, in recent years it has become one of the most important human fungal pathogens in the immunocompromised population [2,31]. Approximately 11 million patients are affected by *Aspergillus* each year. In addition, resistance to existing treatments is emerging [17]. In this study we evaluated the potential of *A*. *fumigatus cpsA* as a possible target for new future strategies against fungal infection. *cpsA* putative homologs have also been found in the pathogenic Basidiomycete *Cryptococcus neoformans*, Cps1, where this gene also positively regulates virulence [40]. In *C*. *neoformans*, Cps1 was experimentally described as a hyaluronic acid synthase [29]. In Ascomycetes, a Cps1 putative homolog was also found in *Neurospora crassa* by Fu et al. [30], however, after extensive chemical analyses hyaluronic acid was not detected in *N*. *crassa* nor in the model fungus *A*. *nidulans* [30,19]. It is possible that this gene was evolutionarily diverted in Ascomycetes to synthesize a different polysaccharide which is of great relevance in the biology of these organisms. Future research efforts will continue to elucidate the identity of this compound in Aspergillus.

Similarly to the case in *A*. *nidulans*, *A*. *fumigatus cpsA* positively affects vegetative growth; deletion of *cpsA* resulted in smaller colonies. However, differently from *A*. *nidulans*, overexpression of *cpsA* in *A*. *fumigatus* resulted in larger colonies when compared to the wild type. In addition, we observed that either deletion or overexpression of *cpsA* caused a drastic reduction in conidial production in both fungi, suggesting that wild-type CpsA levels in balanced stoichiometry with other unknown factors are required for normal conidiation in this fungus. Furthermore, the *brlA*, and *abaA* regulatory genes, which are part of the central developmental pathway [34,41] are *cpsA*-dependent. Specifically, a decrease in *brlA* and *abaA* expression was observed in the *cpsA* deletion mutant compared to the wild type. Interestingly, overexpression of *cpsA* showed an increase in the expression of both *brlA* and *abaA* after 72 h of incubation with respect to the control, while conidial production remained lower than the wild type, suggesting that the effect of *cpsA* on conidiation is complex and involves additional factors in *A*. *fumigatus*. The positive effect of *cpsA* on conidiation appears to also be conserved in *N*. *crassa* [30]. It is likely that other *cpsA* homologs across fungal genera could have a similar effect on development.

As in the case of *N*. *crassa* and *A*. *nidulans* [30,19], *A*. *fumigatus cpsA* was also found to influence cell wall function, as shown by the decrease in growth or development observed when strains lacking or overexpressing *cpsA* were exposed to the perturbing cell wall agents SDS and NZ. Furthermore, we found that *cpsA* affects cell wall composition in *A*. *fumigatus*; deletion of *cpsA* increased level of mannoprotein and insoluble glucan when compared to the isogenic control strain. It is possible that the absence of *cpsA* alters the balance between cell wall polysaccharides, activating compensatory mechanisms [42,10,43]. These results differ from those in *A*. *nidulans* studies where *cpsA* deletion and overexpression exhibited a decrease in mannoprotein, soluble and insoluble glucan, and also chitin [19], suggesting that *cpsA* might not be functionally identical in *A*. *nidulans* and *A*. *fumigatus*.

Biofilm protects the fungus from external stresses, including antifungal agents [44]. Adherence is a requirement for biofilm formation [11]. Our results showed that *A*. *fumigatus cpsA* also influences adhesion to surfaces. Both, deletion or overexpression of *cpsA* resulted in a decrease in adhesion capacity. This effect is similar to that observed in *A*. *nidulans* [19], however, loss of adherence in the absence of *cpsA* was more drastic in the model fungus than in *A*. *fumigatus*.

Cell wall composition and biofilm formation are important in pathogenicity [45,46]. Cell wall alterations and the reduction of adhesion capacity in the *cpsA* deletion mutant could have contributed to the significant decrease in virulence in our murine model, specifically in the non-neutropenic environment. In addition, these alterations in the *cpsA* mutant could have also caused the observed greater sensitivity to oxidative stress, which could further weaken virulence during infection. Resistance to oxidative stress is relevant when fungal cells are exposed to immune system components such as macrophages and neutrophils, cell types present in non-neutropenic patients. These immune cells use reactive oxygen species (ROS) as one of the armors in controlling invading pathogens during immune clearance [47]. In our pathogenicity study using non-neutropenic mice, ROS may have been more efficient against the *A*. *fumigatus* strain lacking *cpsA* than against the wild type.

Other factors that influence virulence include the production of fungal secondary metabolites. Previously, in *A*. *nidulans*, *cpsA* was shown to positively regulate the production of the mycotoxin sterigmatocystin as well as the antibiotic penicillin [19]. For this reason we analyzed the *cpsA*-dependent metabolome in *A*. *fumigatus*. In this fungus, deletion of *cpsA* had a limited effect on secondary metabolism under the experimental conditions assayed. Our results suggest that the observed reduction in virulence in the absence of *cpsA* might not be a consequence of *cpsA*-dependent alterations of secondary metabolism. However, the role of *cpsA* on the production of these compounds in the host environment could be greater than that observed in laboratory cultures, affecting virulence. Future metabolomics studies of infected host tissue will address this possibility.

In addition, overexpression of *cpsA* resulted in a decrease in the production of fumigaclavine A, fumigaclavine B and fumigaclavine C. Interestingly, when *cpsA* was overexpressed we observed significant increases in the production of compounds with potential biotechnological applications, such as verruculogen TR-2 and tryprostatin A. Verruculogen TR-2 presents antifungal and antifeedant properties, and the diketopiperazine tryprostatin A has manifested anti-tumoral properties inhibiting the M phase of the mammalian cell cycle [48,49].

In conclusion, this study shows that *cpsA* is a positive regulator of *A*. *fumigatus* pathogenesis in a non-neutrophenic murine model, influencing cellular processes that affect tissue colonization of this opportunistic pathogen, such as adhesion, cell wall composition and oxidative stress resistance. In addition, *cpsA* affects fungal growth rate and development, which could further affect virulence, as well as dissemination and survival of *A*. *fumigatus*, making *cpsA* or its gene product, CpsA, potential targets in the fight against Aspergillosis. *cpsA* is absent in the human genome; however, it is found in other fungi, and therefore it is likely that a safe control strategy using *cpsA* could be effective in the treatment of fungal infections caused by *A*. *fumigatus* and possibly by other pathogenic species. In addition, forced overexpression of this gene results in an increase in the production of bioactive compounds with potential biotechnological applications.

## Supporting information

S1 Fig Generation of *A*. *fumigatus cpsA* deletion strain (Δ*cpsA*), complementation strain (Com) and overexpression strain (OE) strain. (A) Schematic diagram and image showing the replacement of *cpsA* with the *A*. *parasiticus pyrG* gene (*pyrG*^*A*. *para*^) by a double-crossover event. *Kpn*I restriction sites and probe template used in the Southern blot analysis to confirm the proper integration of the cassette are shown. The probe was PCR amplified with primers cps5F and cps5R (Table 2). The expected band sizes were 5.6 kb for wild type (WT) and 2.1 kb for Δ*cpsA*. (B) Generation of Com strain. Schematic diagram of complementation plasmid is shown. Confirmation of transformants was carried out with diagnostic PCR using primers 1918F and 1986R. The expected band size of 2.1 kb was obtained. Wild-type genomic DNA and plasmid vector pJET+*cpsA* (*cpsA* wild-type allele ligated to the commercial pJET vector—Thermo Scientific) were used as positive controls, Δ*cpsA* genomic DNA was used as a negative control. (C) Generation of OE strain. Schematic diagram of overexpression plasmid is shown. Confirmation of transformants was also done by diagnostic PCR using primers 592F and 1927R. The expected band size of 2.2 kb was obtained. The overexpression vector pBN2 was used as positive control and wild-type genomic DNA was used as a negative control.
(PDF)

S2 Fig *cpsA* is dispensable for resistance to osmotic stress. GMM supplemented with different osmotic stabilizer was point-inoculated with *A*. *fumigatus* wild type, Δ*cpsA*, Com and OE strains and incubated at 37 °C for 72 h. Arrow indicates an unknown pigment produced by Δ*cpsA* in GMM plus 0.8M Sucrose or 1.2 M Sorbitol.
(PDF)
