# Supplementary figures and images for: Characterization of the putative polysaccharide synthase CpsA and its effects on the virulence of the human pathogen Aspergillus fumigatus
Source: PLoS One. 2019 Apr 26;14(4):e0216092. doi: 10.1371/journal.pone.0216092 (PMC6485754; doi:10.1371/journal.pone.0216092)

**A**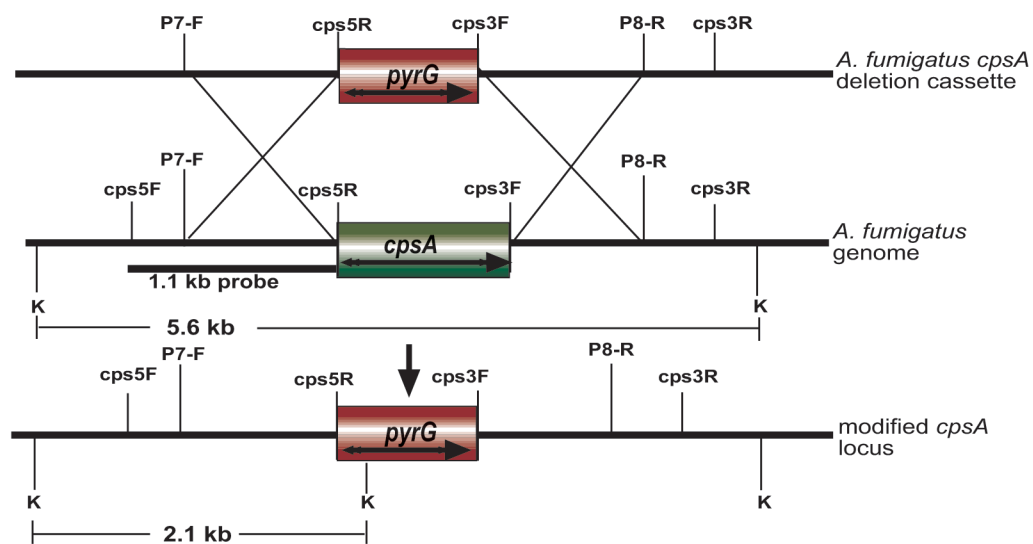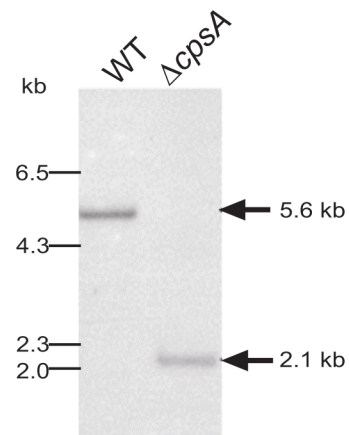**B**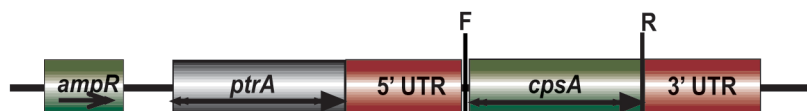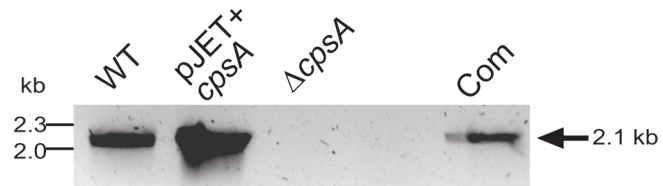**C**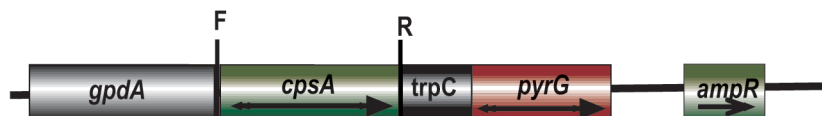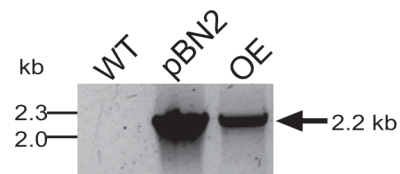

Supplement: S1 Fig — (A) Schematic diagram and image showing the replacement of cpsA with the A. parasiticus pyrG gene (pyrGA. para) by a double-crossover event. KpnI restriction sites and probe template used in the Southern blot analysis to confirm the proper integration of the cassette are shown. The probe was PCR amplified with primers cps5F and cps5R (Table 2). The expected band sizes were 5.6 kb for wild type (WT) and 2.1 kb for ΔcpsA. (B) Generation of Com strain. Schematic diagram of complementation plasmid is shown. Confirmation of transformants was carried out with diagnostic PCR using primers 1918F and 1986R. The expected band size of 2.1 kb was obtained. Wild-type genomic DNA and plasmid vector pJET+cpsA (cpsA wild-type allele ligated to the commercial pJET vector—Thermo Scientific) were used as positive controls, ΔcpsA genomic DNA was used as a negative control. (C) Generation of OE strain. Schematic diagram of overexpression plasmid is shown. Confirmation of transformants was also done by diagnostic PCR using primers 592F and 1927R. The expected band size of 2.2 kb was obtained. The overexpression vector pBN2 was used as positive control and wild-type genomic DNA was used as a negative control. (PDF) [file pone.0216092.s001.pdf]

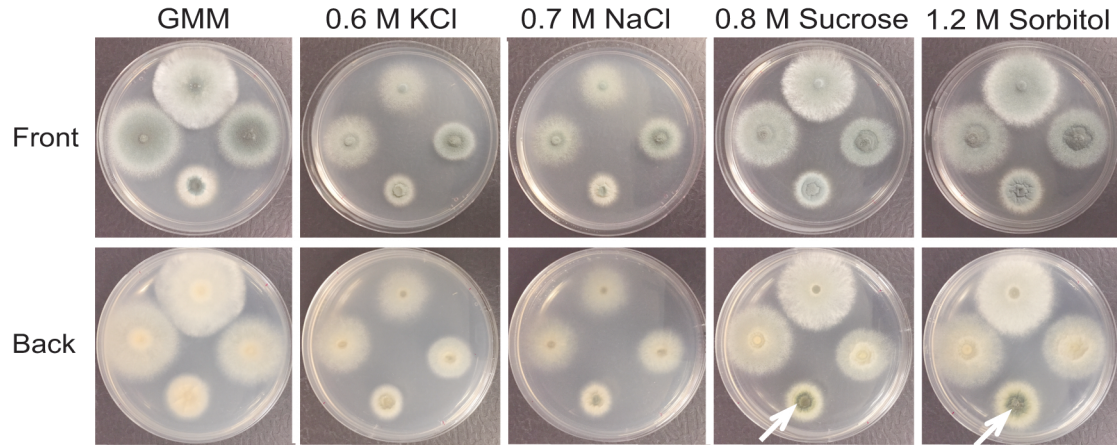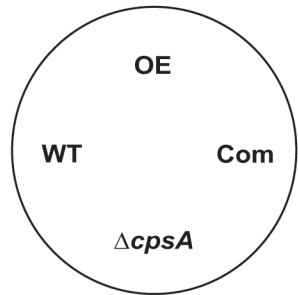

Supplement: S2 Fig — GMM supplemented with different osmotic stabilizer was point-inoculated with A. fumigatus wild type, ΔcpsA, Com and OE strains and incubated at 37 °C for 72 h. Arrow indicates an unknown pigment produced by ΔcpsA in GMM plus 0.8M Sucrose or 1.2 M Sorbitol. (PDF) [file pone.0216092.s002.pdf]
